# Supplementary material for: Austrian Veterinarians’ Attitudes to Euthanasia in Equine Practice
Source: Animals (Basel). 2019 Jan 30;9(2):44. doi: 10.3390/ani9020044 (PMC6406998; doi:10.3390/ani9020044)
Supplement: Supplementary file 1 [file animals-09-00044-s001.zip › Supplementary file 1 and 2/Supplementary_File_1_Questionnaire_German.pdf]

Euthanasie in der Pferdepraxis

Umfrage zur Euthanasie in der Pferdemedizin

Liebe Kolleginnen und Kollegen aus der tierärztlichen Praxis,

das Thema „Tötung von Tieren" spielt in der gesellschaftlichen Wahrnehmung eine zunehmend wichtige Rolle. Der Wandel der Mensch-Tier-Beziehung konfrontiert praktische Tierärztinnen und Tierärzte im Berufsleben mit neuen Herausforderungen. Sie haben in Ihrem Berufsleben professionelle Erfahrungen gemacht, die für angehende Tierärztinnen und Tierärzte von großem Wert sein können. Wir möchten daher mit Ihrer Hilfe und dem folgenden Fragebogen ein Bild der Ansichten und Einstellungen zum Thema „Tötung von Pferden" erstellen: Was sind die prägenden Einstellungen? Was macht die Euthanasie zu (k)einem Problem? Was ist die Rolle der Tierärztinnen und Tierärzte? Welche Strategien sind Ihnen wichtig? Welche Tipps können Sie weitergeben?

Dieses interdisziplinäre Projekt wird im Rahmen einer Diplomarbeit von Magdalena Weber-Schallauer unter der Leitung von Herrn Prof. Herwig Grimm (Messerli Forschungsinstitut) und Frau Prof. Florien Jenner (Pferdeklinik der Veterinärmedizinischen Universität Wien) durchgeführt. Der Fragebogen baut auf einer Umfrage zur Praxis der Euthanasie in der Kleintiermedizin auf und wurde in Zusammenarbeit mit der Studienautorin Svenja Springer in der Arbeitsgruppe „*Ethik in der Pferdeklinik*" an der Veterinärmedizinischen Universität Wien für die Pferdepraxis erarbeitet.

Ziel ist es, die Ergebnisse auch dafür zu verwenden, angehende Tierärztinnen und Tierärzte besser auf den Berufsalltag vorzubereiten. Die Ergebnisse der Befragung werden Ihnen über die Homepage des Messerli Forschungsinstitutes zugänglich gemacht beziehungsweise in geeigneter Form publiziert.

Ihre Angaben werden selbstverständlich vertraulich behandelt und anonymisiert ausgewertet.

Diese Umfrage enthält 57 Fragen und wird eine gewisse Zeit in Anspruch nehmen. Wir bitten Sie dennoch sich diese zu nehmen. Ihre praktische Erfahrung, Ihre Expertise und Ihrer persönlichen Ansichten und Einstellungen stellen eine wichtige und wertvolle Bereicherung für die Erforschung des Themas dar.

Entsprechend zielen die gestellten Fragen auf Ihre persönliche Einstellung. Es geht nicht um die Auskunft zu den rechtlichen Vorgaben, sondern um eine Ermittlung der vorherrschenden moralischen Einstellungen und Haltungen in Ihrem Praxisbereich.

---

[11] Fragebogen aus GoogleFormular exportiert und für Microsoft Word adaptiert; vorliegende Version ohne Antwortskalen.

Fallbeispiele

Im folgenden Abschnitt geht es um Ihre Einstellung zur Euthanasie. Wir stellen Ihnen verschiedene Fallbeispiele vor und bitten Sie, uns Ihre persönliche Sicht mitzuteilen. Zur Beantwortung der einzelnen Fragen finden Sie eine Skala von 1 bis 9. Dabei bedeutet 1, dass Sie die Euthanasie sicherlich ablehnen, und 9, dass Sie der Euthanasie sicherlich zustimmen.

1 [F1]

Ein Besitzer wendet sich mit seiner hochgradig an Hufrehen erkrankten Stute an Sie. Sie wissen, dass dieser eine sehr enge Bindung zu seinem Pferd hat und sich nicht von diesem trennen möchte. Aus Ihrer Sicht ist eine Euthanasie eindeutig angezeigt. Nach einem ausführlichen Aufklärungsgespräch möchte der Tierbesitzer dennoch die Stute am Leben erhalten.

2 [F2]

Ein Besitzer kommt mit einer 15-jährigen Stute zu Ihnen. Die Stute hat eine frische Wunde in der linken vorderen Fesselbeuge mit Beteiligung des Fesselgelenks. Sie geben dem Pferd eine gute Prognose für die Verwendung als Freizeitreitpferd. Der Tierbesitzer lehnt die Therapie aus Kostengründen ab und möchte, dass Sie das Tier euthanasieren.

3 [F3]

Eine Besitzerin von zwei Islandpferden stirbt und vermacht ihre beiden Tiere einem Freund zur weiteren Betreuung. Die Pferde wurden seit Fohlenalter und über mehr als 30 Jahre zusammen gehalten. Eines Tiere stirbt im Alter von 37 Jahren. Im Testament der ehemalige Besitzerin folgendes verfügt: Im Falle des Todes eines der beiden Pferde, soll das andere euthanasiert werden, um dem hinterbliebenen Pferd das Leid durch die Trennung zu ersparen. Das hinterbliebene Pferd ist für sein Alter in einem ausgezeichneten Zustand und es spricht nichts dagegen, dass es noch einige Jahre zu leben hat. Der neue Besitzer bittet Sie, das hinterbliebene Pferd zu euthanasieren.

4 [F4]

Ein Besitzer eines jungen Hengstes wendet sich an Sie. Der Hengst hat eine komplizierte Rissquetschwunde am linken Hinterbein. Sie geben dem Pferd eine sehr gute Prognose für volle gesundheitliche Wiederherstellung. Der Tierbesitzer lehnt die Therapie aus

Sorge ab, dass die erforderliche Boxenruhe von etwa 12 Wochen das Wohlbefinden des Hengstes massiv einschränkt und möchte, dass Sie den Hengst euthanasieren.

5 [F5]

Sie werden zu einer Stute mit Fohlen gerufen. Das Fohlen zeigt Symptome einer schweren Kolik. Die Züchterin möchte die vorgeschlagene OP nicht durchführen lassen, da diese sie mehr kosten würde, als sie durch den Verkauf des Fohlens verdienen würde. Die Patientenbesitzerin möchte, dass Sie das Fohlen euthanasieren.

6 [F6]

Sie erhalten einen Anruf von einer Frau, die auf die 21-jährige Stute eines Paares aufpasst. Das Paar ist vor zwei Tagen zu einer vierwöchigen Trekkingtour aufgebrochen und nicht erreichbar. Seit sechs Monaten behandeln Sie das Pferd wegen chronischer Bronchitis. Nach anfänglich gutem Ansprechen auf die Therapie, geht es dem Pferd jetzt massiv schlechter und es spricht nicht mehr auf die Behandlung an. Die Frau weigert sich, eine Entscheidung bezüglich Euthanasie zu treffen und kann Ihnen auch keine Auskunft darüber geben, was die Besitzer vermutlich wollen würden.

7 [F7]

Eine Pferdebesitzerin kommt mit dem Anliegen ihren 25-jährigen Wallach zu euthanasieren. Die Begründung lautet, dass er nicht mehr zu ihren Lebensumständen passt, da sie mit ihrer Familie für längere Zeit verreist und das Pferd in diesem Alter nicht in fremde Obhut in einem anderen Stall geben möchte.

weitere Fallbeispiele

Bei den folgenden Fallbeispielen möchten wir Sie bitten, dass Sie uns mithilfe der Antwortskala antworten.

8 [F8]

Ihnen wird eine 25-jährige Stute vorgestellt. Aufgrund des Altersgebisses ist sie nicht mehr fähig Raufutter aufzunehmen. Sie ist bereits deutlich abgemagert und zeigt kein Interesse an ihrer Umgebung. Aufgrund des schlechten Allgemeinzustandes des Tieres, sind Sie der Meinung, dass die Prognose infaust ist und raten dem Besitzer zu einer

Euthanasie. Der Tierbesitzer möchte das Tier nicht euthanasieren lassen. Sind Sie der Meinung, dass der Amtstierarzt in diesem Fall, wenn das Tier nicht euthanasiert wird, informiert werden muss.

Bitte kreuzen Sie die zutreffende Antwort auf der Skala an (1 = Informieren des Amtstierarztes ablehnen; 9 = Informieren des Amtstierarztes zustimmen).

9 [F9]

Ein 12-jähriger Warmblutwallach mit einer geschlossenen Fraktur des Fesselbeins wird eingeliefert. Sie geben dem Pferd eine 50%ige Chance auf Überleben nach chirurgischer Intervention. Die Besitzer sind unentschlossen und fragen Sie: „Was würden Sie tun, wenn es Ihr Tier wäre?" Würden Sie eine persönliche Empfehlung abgeben und damit eventuell die Entscheidung übernehmen?

Bitte klicken Sie Ihre Antwort auf der Skala an (1 = ich würde keine Empfehlung abgeben, 9 = ich würde eine Empfehlung abgeben).

10 [F10]

Sie werden zu einem Shetlandpony in Seitenlage gerufen, das kurz davor von einem PKW angefahren wurde. Das Pony ist apathisch. Sie stellen während der Untersuchung fest, dass das Pony aufgrund der Unfallverletzungen nicht mehr am Leben zu erhalten ist und schlagen den Besitzern eine Euthanasie vor. Die Besitzer entscheiden sich für die Euthanasie. Würden Sie das Pony trotz seines apathischen Zustandes vor der Administration des Euthanasiepräparates sedieren?

Bitte beantworten Sie diese Frage mithilfe der Antwortskala (1 = „Ich würde das Pony sicherlich sedieren", 9 = „Ich würde das Pony sicherlich nicht sedieren").

Aussagen und Statements I

In den folgenden drei Abschnitten finden Sie eine Reihe von Aussagen und Statements, die wir in der Literatur gefunden oder in Gesprächen mit Tierärzten und Tierärztinnen gehört haben. Einige der Aussagen beziehen sich ganz allgemein auf den Umgang mit der Euthanasie und Umstände, die den Umgang damit erleichtern oder erschweren. Andere Aussagen beziehen sich eher auf die praktische Gestaltung und Durchführung.
Zur Beantwortung der einzelnen Fragen finden Sie eine Skala von 1 bis 9. Dabei bedeutet 1, dass Sie der Aussage überhaupt nicht zustimmen, und 9, dass Sie der Aussage völlig zustimmen.

Hier ist der erste Abschnitt.

11 [AI1]

Das Wissen, mich nach bestem Wissen für das Wohl des Pferdes eingesetzt zu haben, macht es mir leichter, mit dem Leiden des Pferdes umzugehen, wenn keine Zustimmung des Besitzers zur Euthanasie vorliegt.

12 [AI2]

Das Wissen, dass das Pferd nur noch eine kurze Lebensspanne vor sich hatte, erleichtert mir das Akzeptieren einer medizinisch indizierten Euthanasie.

13 [AI3]

Die sorgfältige Aufklärung des Pferdebesitzers macht es mir leichter, mit der Durchführung einer Euthanasie umzugehen.

14 [AI4]

Die Einsicht des Besitzers, dass die Euthanasie seines Pferdes notwendig ist, macht es mir leichter, mit der Euthanasie umzugehen.

15 [AI5]

Das Wissen, dass alle veterinärmedizinischen wie auch sozialen und ökonomischen Aspekte bei der Entscheidung für eine Euthanasie berücksichtigt wurden, macht es mir leichter mit der Euthanasie umzugehen.

16 [AI6]

Es fällt mir leichter, ein Pferd zu euthanasieren, wenn ich den Eindruck habe, dass die Tierbesitzer keine intensive Bindung zu dem Tier haben.

17 [AI7]

Wenn die Pferdebesitzer bei der Euthanasie anwesend sind, erleichtert mir dies, mit der Euthanasie umzugehen.

18 [AI8]

Generell fällt es mir immer noch schwer, Pferde zu euthanasieren.

19 [AI9]

Die sorgfältige Planung und die gezielte Wahl des Zeitpunktes machen es mir leichter, mit der Euthanasie umzugehen.

20 [AI10]

Die technisch einwandfreie Durchführung und ein komplikationsloser Ablauf der Tötung des Tieres macht es mir leichter, mit der Euthanasie umzugehen.

Aussagen und Statements II

Hier beginnt der zweite Abschnitt.

21 [AII1]

Eine wirksame Schmerztherapie macht es mir leichter, mit dem Leid des Pferdes umzugehen.

22 [AII2]

Die sorgfältige Aufklärung des Patientenbesitzers macht es mir leichter, mit dem Leid des Tieres umzugehen, wenn sich die Besitzer gegen eine Euthanasie des Pferdes entscheiden.

23 [AII3]

Der Eindruck, dass ein Tier bis zum Zeitpunkt der Euthanasie ein erfülltes Leben hatte, macht es mir leichter mit der Euthanasie umzugehen.

24 [AII4]

Belastend wäre es für mich, ein Pferd gegen die eigene Überzeugung zu euthanasieren.

25 [AII5]

Die Anwesenheit des Pferdebesitzers während der Euthanasie verursacht tendenziell mehr Probleme.

26 [AII6]

Der respektvolle Umgang mit dem toten Pferd ist ein wichtiger Teil der Euthanasie.

27 [AII7]

Der verständnisvolle Umgang mit den Pferdebesitzern ist ein zentraler Bestandteil der Euthanasie.

28 [AII8]

Mit zunehmender Berufserfahrung fällt es mir leichter mit der Euthanasie eines Pferdes umzugehen.

Aussagen und Statements III

Nun der dritte Abschnitt.

29 [AIII1]

Obwohl ich eine Euthanasie eigentlich ablehnen würde, führe ich sie dennoch auf Wunsch des Besitzers durch, da ich befürchte, dass der Tierbesitzer sein Pferd ansonsten vernachlässigt.

30 [AIII2]

Das Wissen, mich für das Wohl des Tieres eingesetzt zu haben, macht es mir leichter, mit der Euthanasie umzugehen.

31 [AIII3]

Ich erlebe Euthanasie als unvermeidliches Übel meiner beruflichen Verantwortung.

32 [AIII4]

Die Einsicht, dass die Möglichkeiten meiner Einflussnahme auf die Entscheidung des Besitzers begrenzt sind, macht es mir leichter, mit der Euthanasie umzugehen.

33 [AIII5]

Fortgeschrittenes (hohes) Alter eines Pferdes macht es mir leichter, mit dessen Euthanasie umzugehen.

34 [AIII6]

Obwohl ich eine Euthanasie eigentlich ablehnen würde, führe ich sie dennoch durch, wenn ich befürchte, dass sich der Pferdebesitzer sonst an einen anderen Tierarzt wendet.

35 [AIII7]

Ich sehe die wohlüberlegte Euthanasie als positiven Bestandteil meiner tierärztlichen Tätigkeit an.

Allgemeine und demografische Fragen

Im letzten Abschnitt haben wir noch ein paar demografische Fragen zu Ihnen, Ihrer beruflichen Tätigkeit und zur Euthanasie.

36 [D1] Wie groß ist der Pferdeanteil Ihrer Patienten?

Bitte wählen Sie die zutreffende Antwort aus:

< 20 %	20 - 40 %	41 - 60 %	61 - 80 %	> 80 %
○	○	○	○	○

37 [D2] Sollten unter 80% Ihres Patientenaufkommens Pferde sein, in welchem veterinärmedizinischen Bereich sind Sie überwiegend noch tätig?

Bitte wählen Sie die zutreffende Antwort aus:

○ **Kleintiere**

○ **Landwirtschaftliche Nutztiere**

Andere:_____________

38 [D3]

Sind Sie selbstständig in Ihrem Beruf tätig oder arbeiten Sie im Angestelltenverhältnis in einer Klinik/Praxis?

Bitte wählen Sie die zutreffende Antwort aus:

selbständig	angestellt
○	○

39 [D4]

Wie viele Tierärzte und/oder Tierärztinnen sind außer Ihnen in Ihrer Klinik/Praxis beschäftigt?

Bitte geben Sie hier die Anzahl der Tierärzte/Tierärztinnen an, die ebenfalls in Ihrer Klinik/Praxis tätig sind.

40 [D5] Wie viele Pferde werden in Ihrer Klinik/Praxis pro Monat durchschnittlich euthanasiert?

Bitte geben Sie die durchschnittliche Anzahl der monatlich euthanasierten Pferde an.

41 [D6] Wie häufig pro Monat euthanasieren Sie selbst?

Bitte geben Sie hier an, wie häufig Sie pro Monat selbst euthanasieren.

42 [D7]

Wie häufig werden Sie pro Jahr in etwa ersucht, ein Pferd entgegen Ihrem Rat / Ihrer Überzeugung zu euthanasieren?

Bitte geben Sie hier die Häufigkeit pro Jahr an.

43 [D8]

Wie häufig werden Sie pro Jahr schätzungsweise ersucht, ein (weitgehend) gesundes Pferd zu euthanasieren?

Bitte geben Sie hier die Häufigkeit pro Jahr an.

44 [D9]

Wenn Tierhalter ein (weitgehend) gesundes Pferd euthanasieren lassen möchten, welche sind die drei am häufigsten genannten Gründe?

Bitte geben Sie Ihre Antwort(en) hier ein:

- 1. häufigster Grund

- 2. häufigster Grund

- 3. häufigster Grund

45 [D10]

Welches Euthanasiepräparat verwenden Sie in Ihrer Ordination für die Euthanasie von Pferden? (Mehrfachantwort möglich)

Bitte wählen Sie alle zutreffenden Möglichkeiten aus und schreiben Sie gerne auch einen Kommentar dazu:

- ☐ Kombinationspräparate mit Embutramid (z.B. T61®)

- ☐ Derivate der Barbitursäure (z.B. Thiopental, Pentobarbital)

- ☐ physikalische Euthanasie (z.B. Erschießen)

- ☐ andere:

46 [D11] Verwenden Sie immer schon denselben Wirkstoff zur Euthanasie?

Bitte geben Sie Ihre Antwort hier ein:

47 [D12] Falls Sie den Wirkstoff zur Euthanasie schon einmal gewechselt haben, was war der Grund dafür?

Bitte geben Sie Ihre Antwort hier ein:

48 [D13] Was sind die drei am häufigsten auftretenden Komplikationen bei der Durchführung einer Euthanasie?

- 1.:
- 2.:
- 3.:

49 [D14] Wie viele der von Ihnen durchgeführten Euthanasien laufen in etwa komplikationslos ab?

Bitte geben Sie Ihre Antwort hier in Prozent ein:

- _____%

50 [D15] Wie viele Jahre sind Sie bereits als PferdepraktikerIn tätig?

Bitte geben Sie hier an, wie viele Jahre Sie bereits tierärztlich tätig sind.

51 [D16] Bitte sagen Sie uns auch, wie alt Sie sind.

Bitte geben Sie Ihr Alter in Jahren an.

52 [D17] Bitte geben Sie hier Ihr Geschlecht an.

Bitte wählen Sie die zutreffende Antwort aus.

männlich ○
weiblich ○

53 [D18] Was (oder wer) hat Sie fachlich am besten für Ihre beruflichen Aufgaben im Bereich der Euthanasie vorbereitet?

Bitte geben Sie Ihre Antwort hier ein:

54 [D19] Was oder wer hilft Ihnen heute eine Euthanasie psychisch zu verarbeiten?

Bitte geben Sie Ihre Antwort hier ein:

55 [D20] Würden Sie sich mehr Unterstützung hinsichtlich der Thematik Euthanasie wünschen, und wenn ja von wem?

Bitte geben Sie Ihre Antwort hier ein:

56 [D21]

Würde Ihnen in schwierigen Situationen ein Kriterienkatalog zur Unterstützung bei der Entscheidung für oder gegen eine Euthanasie helfen?

Bitte wählen Sie die zutreffende Antwort aus:

Ja	Unsicher	Nein
○	○	○

57 [D22] Gibt es weitere Kommentare Ihrerseits zum Thema Euthanasie oder zum Fragebogen?

Bitte geben Sie Ihre Antwort hier ein:
