# Supplementary material for: Austrian Veterinarians’ Attitudes to Euthanasia in Equine Practice
Source: Animals (Basel). 2019 Jan 30;9(2):44. doi: 10.3390/ani9020044 (PMC6406998; doi:10.3390/ani9020044)
Supplement: Supplementary file 1 [file animals-09-00044-s001.zip › Supplementary file 1 and 2/Supplementary_File_2_Questionnaire_English.pdf]

Euthanasia in Equine Practice

English version of the questionnaire on euthanasia in equine practice

Case Scenarios

For the scenarios F1-F7, the veterinarians were asked “What is your attitude towards euthanasia?” and had the possibility to gauge their agreement with euthanasia in this case from 1=” I reject euthanasia” to 9=”I fully agree with euthanasia”. In scenario F8 the question was about the necessity to notify an official veterinarian, with the answer options ranging from 1=”rejection” to 9=”agreement”. The answer options for scenario F9, asking about the willingness to take decision concerning euthanasia in the place of the owner, ranged from 1=”I am sure I would make no recommendation” to 9=”I am sure I would make a recommendation”. Scenario F10 includes a technical aspect and veterinarians had the possibility to choose from 1=”I am sure I would sedate the animal” to 9=”I am sure I would not sedate the animal”.

1 [F 1]
An owner turns to you with his severely laminitic mare. You know that he is very attached to his horse and does not want to part with it. From your point of view, euthanasia is clearly indicated. After a detailed explanation, the owner still refuses euthanasia.

2 [F 2]
A 15-year-old mare with a fresh wound with involvement of the left front fetlock joint is presented to you. You give the horse a good prognosis for future use as a leisure riding horse. The animal owner refuses therapy for financial reasons and wants you to euthanise the animal.

3 [F 3]
An owner of two Icelandic horses dies and bequeaths her two animals to a friend for further care. The horses have been kept together since they were foals, for more than 30 years. One of the animals dies at the age of 37. In the will of the deceased owner, the following is stipulated: In the event of the death of one of the two horses, the other is to be euthanised in order to save the surviving horse from suffering through separation. The surviving horse is in excellent condition for its age and there is no reason why it should not live for a few more years. The new owner asks you to euthanise the surviving horse.

4 [F 4]
The owner of a young stallion contacts you. The stallion has a complicated contusion laceration of the left hind leg. You give the horse a very good prognosis for full recovery. The animal owner refuses therapy because he is concerned that the stallion could not cope with stall confinement for the required 12-week period. He

believes it would affect the horse's quality of life to the point that he would like you to euthanise the stallion.

5 [F 5]
You are called to a mare with a foal. The foal shows symptoms of severe colic. The owner does not want to have the proposed operation carried out, as the costs would exceed the foal's monetary value, and wants you to euthanise the foal.

6 [F 6]
You receive a phone call from a woman who looks after the 21-year-old mare of friends who just left for a four-week trekking tour and are not reachable. You have been treating the horse for six months for chronic bronchitis. The horse initially responded well to therapy is now deteriorating and does not respond to treatment anymore. The woman refuses to make a decision regarding euthanasia and cannot tell you what the owners might want.

7 [F 7]
A horse owner asks you to euthanise her 25-year-old gelding because she intends to travel with her family for an extended period of time and does not want to uproot the horse at his age by moving him to a boarding stable.

8 [F 8]
You are presented with a 25-year-old mare which appears to be severely neglected, is already highly cachectic and shows no interest in her surroundings. Due to the poor general condition of the animal, you believe that it is already close to death and the prognosis is infaust. Do you think that the official veterinarian should be informed in this case?

9 [F9]
You are presented with a 12-year-old warmblood gelding with a closed fracture of the proximal phalanx. You give the horse a 50% chance of survival with surgical intervention. The owners are indecisive and ask: "What would you do if it was your animal?" Would you make a personal recommendation and thus possibly influence and effect make the decision?

10 [F 10]
You are called to a Shetland pony in lateral recumbency, which was hit by a car. The pony is apathetic. During the examination, you determine that the pony has sustained fatal injuries and recommend euthanasia, which the owners consent to. Would you sedate the pony before administering the euthanasia medication despite its apathetic state?

Statements I

Please indicate your attitude towards each statement using the scale from 1 (“I do not agree at all”) to 9 (“I completely agree”).

11 [AI1]

Knowing that I have done my best for the horse’s well-being makes it easier for me to deal with the horse's suffering if the owner does not agree to euthanasia.

12 [AI2]

It is easier for me to deal with a medically indicated euthanasia if I know that the horse has only a short remaining lifespan.

13 [AI3]

If the horse owner is thoroughly informed I find it easier to deal with euthanasia.

14 [AI4]

The owner's understanding that the euthanasia of his/her horse is necessary makes it easier for me to deal with euthanasia.

15 [AI5]

Knowing that all veterinary medical, social and economic options have been considered in the decision-making process makes it easier for me to deal with euthanasia.

16 [AI6]

It is easier for me to euthanise a horse if I have the impression that the animal’s owners have no close relationships with the animal.

17 [AI7]

It is easier for me to deal with euthanasia if the owners are present during the procedure.

18 [AI8]

Generally, it is still difficult for me to euthanise horses.

19 [AI9]

Careful planning and the right moment make it easier for me to deal with euthanasia.

20 [AI10]

A technically flawless and uncomplicated euthanasia of the animal makes it easier for me to deal with euthanasia.

Statements II

21 [AII1]
Effective pain therapy makes it easier for me to deal with a horse's suffering.

22 [AII2]
If the patient's owner is thoroughly informed I find it easier to deal with the suffering of the animal, even if the owners decide against euthanasia of the horse.

23 [AII3]
It is easier for me to deal with euthanasia if I have the impression that the animal has lived a rich life until its death.

24 [AII4]
It would be difficult for me to euthanise an animal against my convictions.

25 [AII5]
The presence of the horse owner during euthanasia tends to cause more problems.

26 [AII6]
Treating the dead horse in a respectful way is an important part of euthanasia.

27 [AII7]
Treating the owners with compassion is a central part of euthanasia.

28 [AII8]
With increasing professional experience it becomes easier for me to deal with the euthanasia of a horse.

Statements III

29 [AIII1]
I would euthanise an animal in cases without medical indication for euthanasia if I am afraid that the owner will neglect the horse.

30 [AIII2]
It is easier for me to deal with euthanasia if I know that I have done my best for the animal's well-being.

31 [AIII3]
I see euthanasia as an unavoidable evil of my professional responsibility.

32 [AIII4]
Knowing that my influence on the owner’s decision is limited makes it easier for me to deal with euthanasia.

33 [AIII5]
The animal’s advanced (high) age makes it easier for me to deal with euthanasia.

34 [AIII6]
I would euthanise an animal in cases without medical indication for euthanasia if I am afraid that the owner will see another vet.

35 [AIII7]
I see carefully considered euthanasia as a positive part of my practice as a veterinarian.

General and demographic questions

36 [D1]
How many working hours do you spent with horses?

- <20%
- 20-40%
- 41-60%
- 61-80%
- >80%

37 [D2]
Should less than 80% of your patient be horses, in which veterinary field are you still predominantly active?

- Small animals
- Farm animals
- Others: ______________

38 [D3]
Are you working self-employed or employed?

- Self-employed
- Employed

39 [D4]
How many veterinarians are working in your clinic/practice apart from you?

Please enter your answer.

40 [D5]
How many horses are euthanized on average per month in your clinic/practice?

Please enter your answer.

41 [D6]
How often you have to euthanize a horse per month?

Please enter your answer.

42 [D7]
How often you are you confronted with requests to euthanise a horse against your professional opinion per year?

Please enter your answer.

43 [D8]
How often are you confronted with requests to euthanise a (mainly) healthy horse per year?

Please enter your answer.

44 [D9]
If horse owners want to have a (mainly) healthy horse euthanized, what are the three most frequently cited reasons?

- 1. frequent reason:
- 2. frequent reason:
- 3. frequent reason:

45 [D10]
What euthanasia preparation do you use in your practice for euthanasia of horses? (multiple answer possible)

- combination preparation with embutramide (e.g. T61)
- derivatives of barbituric acid (e.g. sodium thiopental, pentobarbital)
- physical euthanasia (e.g. shooting)
- others:______________

46 [D11]
Do you still use the same active ingredient for euthanasia?

47 [D12]
If you have already changed the active ingredient for euthanasia, what was the reason for it?

Please enter your answer.

48 [D13]
What are the three most common complications of performing euthanasia?

- 1.:
- 2.:
- 3.:

49 [D14]
How many euthanasias are complication-free?

- _____%

50 [D15]
How many years have you been working as equine veterinarian?

Please enter your answer.

51 [D16]
Please provide your age in years.

52 [D17]
Please provide your gender.
- Male:
- Female:

53 [D18]
What (or who) prepared you best for euthanasia in a technical sense?

Please enter your answer.

54 [D19]
What (or who) assists you to psychological process with euthanasia currently?

Please enter your answer.

55 [D20]
Do you wish any support in case of euthanasia, and if so, from who?

Please enter your answer.

56 [D21]
Would a catalogue of criteria be a support in difficult decision-making situations?
- Yes
- No
- Uncertain

57 [D22]
Do you have any further comments on euthanasia or on the questionnaire?

Please enter your answer.
